# Auditory acuity in type 2 diabetes mellitus

**DOI:** 10.4103/0973-3930.45270

**Published:** 2008

**Authors:** Pallavi Panchu

**Affiliations:** Department of Physiology, Sree Mookambika Institute of Medical Sciences, Padanilam, Kulashekaram, Kanyakumari, India

**Keywords:** Case control study, Pure Tone Audiometry, Sensorineural Hearing Loss, type 2 diabetes mellitus (type 2 DM)

## Abstract

**BACKGROUND AND OBJECTIVES:**

The relationship between diabetes and hearing loss has been debated for many years. Hyperglycemia appears to have an effect on hearing loss and the proposed mechanisms are microangiopathy, neuropathy or a combination of both. The objective of this study was to evaluate a cross section of hyperglycemic subjects with age- and sex-matched normoglycemic controls with pure tone audiometry and compare the differences.

**MATERIALS AND METHODS:**

Forty-one type 2 diabetes mellitus subjects and 41 age- and sex-matched normoglycemic controls were subjected to a pure tone audiometric assessment followed by evaluation of their glycemic status and degree of glycemic control. The resulting data was statistically analyzed.

**RESULTS:**

The auditory thresholds in hyperglycemic subjects were higher in all age groups in all the frequencies suggestive of sensorineural hearing loss. The hyperglycemic subjects with poor control of their blood sugar levels (HbA1C > 8%) had elevated auditory thresholds in all the test frequencies. The fasting blood sugar level in hyperglycemic subjects showed a trend towards significant difference at higher frequencies, the postprandial blood sugar levels showed significant differences at higher frequencies. There was no effect of duration of diabetes mellitus on the hearing thresholds in hyperglycemic subjects.

**CONCLUSION:**

Subjects with hyperglycemia have a sensorineural hearing loss when evaluated with a pure tone audiometer in all frequencies than a normoglycemic control group. The study showed that post prandial blood sugar levels and HbA1C levels had a direct bearing on the auditory acuity of the hyperglycemic subjects.

## Introduction

Hearing loss is a functional disability which affects a person's day-to-day activities in subtle ways. Sensorineural hearing loss involving the inner ear and its central connections is irreversible. The prevalence of hearing loss in diabetes has been shown in many studies[1–11] to be moderately high, progressive and bilateral. The predominant mechanism of hearing loss in diabetes appears to be related to microangiopathy of the inner ear.[12] The prevalence of hearing loss in diabetics in Indian population has not been studied extensively.[7]

The present study is undertaken to compare auditory acuity in normoglycemic and hyperglycemic subjects to find out the effect of hyperglycemia on auditory acuity.

Diagnostic audiometry comprises tests that detect conductive and sensorineural hearing losses. Pure tone audiometry involves the estimation of the threshold of hearing for certain standardized stimuli via the air and bone conduction routes.[13] An audiometer, being a fundamental tool in the diagnosis of auditory capacities, has been employed in this study.

### Aim of the study

1. To assess the degree of auditory acuity in type 2 diabetes mellitus patients using pure tone audiometry [evaluating the frequency and intensity of hearing acuity]
2. To compare pure tone audiometric results between diabetic type 2 patients [hyperglycemic group] and a matched control group [normoglycemic group].

### Objectives of the study

1. To record pure tone audiometry in normoglycemic subjects.
2. To record pure tone audiometry in hyperglycemic subjects
3. To make a comparative study of the auditory acuity of type 2 diabetics and normoglycemic subjects.
4. To analyze the effect of age, glycemic status (FBS, PPBS), glycemic control (HbA1C), duration of type 2 diabetes on auditory acuity.

## Materials and Methods

### Source of data

The study was conducted in a sample of 82 subjects in Bangalore. The study was approved by the institutional ethics committee of Bangalore Medical College and Rajiv Gandhi University of Health Sciences, Bangalore, India. They have been divided into two groups of 41 subjects each.

Group 1 comprises of 41 normal, healthy subjects of either sex selected from the patient attenders in Victoria hospital belonging to the ages between 35 and 55 years. Group 2 comprises of 41 type 2 diabetic patients of either sex selected from the Department of Medicine Victoria Hospital, belonging to the ages between 35 and 55 years. The sample size was determined after taking into consideration the methods used in other studies. Many published studies on the prevalence of hearing loss in diabetics used a similar sample size between 20 and 45 diabetic subjects.[1,3,6,7,10,11]

### Method of collection of data

The study comprising of 41 type 2 diabetics and 41 nondiabetics, matched with respect to age and sex are selected based on inclusion and exclusion criteria.

### Inclusion criteria [group 1]

Forty-one normal healthy subjects of either sex between 35 and 55 years who had given written consent and who were nonhypertensive were included.

### Inclusion criteria [group 2]

Forty-one type 2 diabetic patients between the ages 35 and 55 years who had given written consent and who were nonhypertensive were included. Both groups were matched with respect to age and sex.

### Exclusion criteria [group 1]

1. Hypertension
2. Diabetes mellitus
3. History of consumption of ototoxic drugs in past three months.
4. History of ear surgeries performed in the past.
5. History of ear infections in the past.
6. History of recent infections in the nose, throat or ear.
7. Patients having a noise induced hearing loss (as shown by pure tone audiometry at 4000 Hz.)

### Exclusion criteria [group 2]

1. History of consumption of ototoxic drugs in past three months.
2. History of ear surgeries performed in the past.
3. History of ear infections in the past.
4. History of recent infections in the nose, throat or ear.
5. Patients having a noise induced hearing loss (as shown by pure tone audiometry at 4000 Hz.)

### Pure tone audiometer

#### Instrument:

ARPHI [500 MK I] audiometer

Ear phones are used to test hearing by air conduction and a small vibrator placed over the mastoid is used test hearing by bone conduction. All audiometers incorporate a calibration circuit, which allows the output sound level to be set at each frequency. The signals presented to the subject by an audiometer are characterized by its frequency, sound pressure level and wave form which are all controlled.[13]

### Methodology

#### Protocol

All the subjects included in this study are given a prepared questionnaire to answer. This questionnaire was designed to reveal the patients' assessment of hearing ability.

An assessment of the hearings status using a pure tone audiometer [ARPHI 500 MK 1] is done.

### Pure tone audiometry

#### Principle:[13]

An audiometer [ARPHI 500 MK 1] is an electronic device that produces pure tones, the intensity of which can be increased or decreased in 5-Db steps. Air conduction thresholds are measured for tones of 250, 500, 1000, 1500, 2000, 4000 6000 and 8000 Hertz. Bone conduction thresholds and measured for 250, 500, 1000, 1500, 2000, 4000 Hertz. The amount of intensity that has to be raised above the normal level is a measure of the degree of hearing impairment at that frequency. It is charted in form of a graph called the “audiogram.” The thresholds of bone conduction are a measure of the cochlear function. The difference in the thresholds of air and bone conduction (A-B gap) is a measure of a degree of conductive deafness. The audiometer is so calibrated that hearing of a normal person, both of air and bone conduction is at 0 db and there is no A-B gap.

### Methodology of pure tone audiometry

The method is based on American Society for Speech and Hearing Association [ASHA] 1978 guidelines for manual pure tone audiometry (PTA). Masked pure tone audiometry is done if there is a difference of more than 40 dB between air conduction threshold of the test ear and the bone conduction threshold of the opposite ear, or when the air bone gap of the poorer ear under test is more than 10 dB.

### Statistical methods[14,15]

Student t test (unpaired and two tailed) has been used to find the significance of auditory thresholds (dB) between various categories of parameters. Analysis of variance [ANOVA] has been used to find the significance of auditory thresholds in different age groups. The effect size (d) has been used to find the effect of DM on auditory thresholds (dB).

### Statistical software:

The statistical soft ware namely SPSS 11.0 and Systat 8.0 were used for the analysis of the data and Microsoft Word and Microsoft Excel have been used to generate graphs, tables, etc.

## Results

Tables 1 and Table 2 show the age and sex distribution of the control group and the diabetics.

**Table 1:** Age distribution of the control group and the diabetics

Age in years	Controls	Cases
35–40	8 (19.51)	8 (19.51)
41–45	8 (19.51)	8 (19.51)
46–50	8 (19.51)	8 (19.51)
51–55	17 (41.46)	17 (41.46)
Total	41	41

**Table 2:** Sex distribution of the control group and the diabetics

Sex	Controls	Cases
Male	16 (39.02)	16 (39.02)
Female	25 (60.98)	25 (60.98)
Total	41	41

Differences in the fasting blood sugar and post-prandial blood sugar level between the cases and controls were statistically significant at 1% as seen in Table 3.

**Table 3:** Blood glucose levels in cases and controls

Sugar Parameters	Controls (Mean ± SD)	Cases (Mean ± SD)	Significance
FBG	80.00 ± 9.29	164.93 ± 64.51	8.344**
PPBG	109.29 ± 8.82	250.68 ± 92.02	9.794**

+ Near significant, *Significant at 5%,

** Significant at 1%

As shown in Table 4, there was a significant difference in the auditory thresholds at all frequencies from 250 Hz to 8000 Hz between type 2 diabetic subjects and control group and all the hyperglycemic subjects showed sensorineural hearing loss changes on audiogram. The effect size was large to very large. The controls, all had normal hearing thresholds, whereas the cases showed a gradual increase in hearing loss starting at 250 Hz and becoming pronounced as the frequency increased. This difference is highly statistically significant at 1% confidence interval.

**Table 4:** Effect of diabetes mellitus on auditory thresholds in dB

Frequency in Hz	Auditory Thresholds in dB (Mean ± SD): normal threshold is less than or equal to 25 db [WHO]				Effect size (d)
		
	Control (n = 41)	Cases (n = 41)	df	Significance by student t	
At 250	21.59 ± 3.48	29.33 ± 8.27	53.73	5.525**	1.21
At 500	21.77 ± 3.37	31.83 ± 6.85	58.26	8.444**	1.85
At 1000	20.49 ± 3.76	29.21 ± 8.88	53.88	5.789**	1.27
At 1500	19.27 ± 4.65	26.59 ± 8.32	62.76	4.913**	1.08
At 2000	19.63 ± 3.64	29.82 ± 8.78	53.52	6.886**	1.50
At 3000	19.21 ± 4.62	27.59 ± 8.65	61.13	5.468**	1.20
At 4000	20.85 ± 4.62	34.21 ± 9.96	52.90	7.951**	1.70
At 6000	20.37 ± 3.69	36.46 ± 10.98	48.92	8.897**	1.95
At 8000	20.12 ± 3.75	35.24 ± 12.39	47.25	7.476**	1.64

+Near significant, *Significant at 5%,

** Significant at 1%

As shown in Table 5, there was no statistically significant difference in auditory thresholds among type 2 diabetic patients and control group when analyzed according to their age groups, yet all type 2 diabetic patients' auditory thresholds were higher than the control groups' thresholds.

**Table 5:** Auditory thresholds (Db) in age-wise subgroups of diabetics

Frequency (Hz)	Group	Auditory Thresholds in dB (Mean ± SD)				Significance ANOVA
			
		35–40 years	41–45 years	46–50 years	51–55 years	
250	Control	18.75±4.43	21.88 ± 2.22	20.63 ± 4.58	23.24 ± 1.71	0.015*
	Case	23.21 ± 6.07	26.56 ±7.19	31.25 ± 6.94	30.88 ± 7.44	0.073+
500	Control	19.38 ± 3.47	20.31 ± 2.48	21.56 ± 4.22	23.68 ± 2.18	0.007**
	Case	27.55 ± 7.32	29.38 ± 6.38	35.63 ± 6.65	33.24 ± 5.91	0.053+
1000	Control	183.43 ± 4.21	19.69 ±3.88	20.00 ± 5.17	22.06 ± 2.02	0.115
	Case	26.25 ± 11.41	25.93 ± 7.89	32.19 ± 9.01	30.74 ± 7.79	0.347
1500	Control	15.94 ± 4.81	17.19 ± 5.08	19.38 ± 5.13	21.76 ± 2.62	0.009**
	Case	21.87 ± 7.76	25.63 ± 9.61	28.44±8.65	28.38±7.55	0.284
2000	Control	18.13 ± 5.13	17.81 ± 2.48	19.69 ± 3.88	21.17 ± 2.67	0.088+
	Case	25.94 ± 9.06	27.19 ± 9.58	30.31 ± 8.28	32.65 ± 8.07	0.256
3000	Control	15.62 ± 3.47	16.88 ± 5.47	19.69 ± 3.64	21.17 ± 3.62	0.004**
	Case	23.13 ± 10.06	26.87 ± 9.33	29.06±6.80	29.32±8.36	0.388
4000	Control	19.69 ± 5.58	19.06 ± 3.99	20.63 ± 4.58	22.35 ± 2.57	0.208
	Case	31.87 ± 13.01	33.13 ± 8.43	34.69 ± 10.13	35.59 ± 9.62	0.842
6000	Control	18.44 ± 3.99	20.31 ± 4.89	19.06±3.76	21.91±2.26	0.099+
	Case	31.25 ± 12.88	37.50 ± 10.77	39.06 ± 11.25	37.21 ± 10.23	0.509
8000	Control	17.50 ± 4.63	20.00 ± 4.82	18.75 ± 2.99	22.06 ± 1.82	0.017*
	Case	30.31 ± 15.37	39.06 ± 12.24	38.13 ± 12.16	34.41 ± 11.23	0.484

+ Near Significant,

* Significant at 5%,

** Significant at 1%

As shown in Table 6, from a frequency of 250 Hz to 8000 Hz, there was a significant difference between diabetic type 2 patients with good control of their blood sugars [HbA1c values between 6% and 8%] versus type 2 diabetic patients with poor control [HbA1c values greater than 8%]. The number of patients in the good control group was 20 and in the poor control group was 21. The significance levels are at 1%.

**Table 6:** Auditory thresholds (db) in HbA1C-wise subgroups of diabetics

Frequency (Hz)	Auditory Thresholds (dB) (Mean ± SD)		*P* value by student t
		
	HbA1c Good Control 6%-8 % (*n* = 20)	HbA1c Poor Control >8 % (*n* = 21)	
250	25.75 ± 5.91	31.62 ± 7.18	0.010**
500	29.50 ± 4.97	34.44 ± 7.55	0.021**
1000	26.13 ± 6.31	32.78 ± 9.69	0.016**
1500	23.38 ± 5.27	30.00 ± 9.19	0.009**
2000	26.38 ± 7.28	33.78 ± 8.41	0.006**
3000	23.88 ± 6.76	32.42 ± 7.89	0.001**
4000	30.63 ± 8.91	39.44 ± 8.93	0.004**
6000	32.25 ± 9.79	42.08 ± 10.62	0.005**
8000	29.88 ± 11.02	41.94 ± 11.59	0.002**

+Near significant, *Significant at 5%,

** Significant at 1%

Table 7 shows that there was no significant difference in the hearing thresholds between patients with short duration of type 2 diabetes [less than 10 years] versus long duration [greater than 10 years].

**Table 7:** Auditory thresholds (db) in duration-wise subgroups of diabetics

Frequency	Auditory thresholds (dB) (Mean ± SD)		*P* value by student t
		
	Duration of DM <10 years (*n* = 34)	Duration of DM >10 years (*n* = 7)	
250	27.73 ± 7.21	33.57 ± 7.34	0.060+
500	31.10 ± 7.05	35.36 ± 4.66	0.136
1000	28.60 ± 8.90	32.14 ± 8.83	0.343
1500	26.25 ± 8.19	28.21 ± 9.43	0.576
2000	29.56 ± 8.36	31.07 ± 11.07	0.682
3000	27.31 ± 8.42	28.93 ± 10.29	0.658
4000	33.97 ± 9.28	35.36 ± 13.65	0.742
6000	35.36 ± 10.32	40.36 ± 14.03	0.309
8000	34.71 ± 11.99	37.86 ± 14.96	0.547

+ Near significant,

*Significant at 5%, **Significant at 1%

Table 8 shows that there is a trend towards a difference which is noted at higher frequencies (6000 Hz and 8000 Hz) when the effect of fasting blood sugar levels on auditory thresholds is considered.

**Table 8:** Auditory thresholds (db) in FBS-wise subdivisions of diabetics

Frequency (Hz)	Auditory thresholds in dB (Mean ± SD) in different FBS levels				Significance ANOVA
		
	50–100 mg/dl (*n* = 5)	101–150 mg/dl (*n* = 15)	151–200 mg/dl (*n* = 10)	>200 mg/dl (*n* = 11)	
250	29.00 ± 5.76	26.83 ± 8.58	29.72 ± 7.12	30.45 ± 7.23	0.651
500	29.00 ± 3.79	30.67 ± 6.91	32.50 ± 8.82	34.09 ± 5.73	0.475
1000	29.00 ± 8.22	26.33 ± 8.65	31.00 ± 10.68	31.59 ± 7.69	0.441
1500	27.00 ± 4.81	23.50 ± 7.12	29.25 ± 10.54	28.18 ± 8.52	0.329
2000	31.00 ± 6.02	25.66 ± 7.76	32.75 ± 8.37	32.77 ± 10.09	0.138
3000	26.00 ± 2.82	23.83 ± 9.72	30.10 ± 8.22	31.14 ± 7.77	0.125
4000	32.50 ± 7.29	30.17 ± 9.84	38.00 ± 9.92	37.04 ± 10.11	0.175
6000	32.00 ± 9.46	31.83 ± 10.06	39.25 ± 8.97	42.27 ± 11.96	0.059+
8000	33.00 ± 11.51	29.50 ± 11.73	39.25 ± 11.55	40.45 ± 12.29	0.090+

+ Near significant,

*Significant at 5%, **Significant at 1%

Table 9 shows that there is a 5% significant difference at 3000, 4000, and 8000 Hz and a 1% significant difference at 6000 Hz when the effect of post prandial blood sugar levels on the auditory thresholds is considered.

**Table 9:** Auditory thresholds (db) in PPBS-wise subdivisions of diabetics

Frequency (Hz)	Auditory thresholds in dB (Mean ± SD) in different PPBS levels				Significance ANOVA
		
	110–200 mg/dl (*n* = 15)	201–300 mg/dl (*n* = 14)	301–400 mg/dl (*n* = 8)	>400 mg/dl (*n* = 4)	
250	26.00 ± 6.93	30.36 ± 7.81	28.21 ± 6.07	34.37 ± 8.26	0.175
500	29.50 ± 5.61	32.66 ± 7.68	32.81 ± 7.49	35.63 ± 6.25	0.351
1000	26.00 ± 7.12	30.00 ± 10.05	31.25 ± 8.35	34.38 ± 10.68	0.285
1500	23.00 ± 5.84	28.75 ± 9.54	27.19 ± 7.84	31.25 ± 10.51	0.169
2000	25.83 ± 6.66	31.96 ± 8.73	30.63 ± 9.04	35.63 ± 11.96	0.118
3000	22.83 ± 6.81	29.89 ± 8.94	28.13 ± 8.10	36.25 ± 6.61	0.017*
4000	30.17 ± 9.75	35.89 ± 9.18	33.75 ± 9.75	44.38 ± 8.75	0.014*
6000	30.83 ± 10.34	38.75 ± 8.86	35.93 ± 10.60	50.63 ± 7.47	0.006**
8000	28.67 ± 12.20	38.39 ± 10.59	35.63 ± 11.39	48.13 ± 8.98	0.018*

+Near significant,

* Significant at 5%,

** Significant at 1%

## Discussion

The relationship between diabetes mellitus and hearing loss is controversial, primarily because the pathogenic mechanism remains obscure. This is a case control study investigating this relationship using pure tone audiometer.

Diabetes has been shown to affect hearing loss by many studies. Many have tried to identify the cause, and based on their conclusions, the probable mechanisms are microangiopathy of the inner ear, neuropathy of the cochlear nerve, a combination of both, outer hair dysfunction and disruption of endolymphatic potential.

This study demonstrates a significant hearing loss in diabetes in all the frequencies tested. This could be explained by microangiopathy of the vessels to the inner ear as proposed by Wackym.[16]

The effect of age on auditory thresholds in diabetic subjects was found to be clinically and statistically insignificant. Kakarlapudi[4] and Dalton[8] both showed similar findings in their study, but Taylor and Irwin[3] found a correlation between hearing levels and age of the subjects in the diabetic group and concluded that any hearing loss due to diabetes will be additional to that due to age alone. Similar conclusions were drawn by Axelson.[5]

Lack of glycemic control shows a positive correlation with extent of hearing loss when compared to those diabetics with good glycemic control. This is noted in all the frequencies tested. Both Kurien[7] and Lasisi[2] show similar findings. A possible mechanism to explain this observation could be the cumulative effects of advanced glycation end products on the inner ear. High post-prandial blood sugar levels cause a significant alteration in high frequency hearing thresholds in diabetics. Damage to outer hair cells by sustained hyperglycemia has been noted in animal studies.[6,17–19] Currently, outer hair cell function in diabetes is an area of intense research activity.

The present study supports the hypothesis that duration of diabetes does not alter hearing thresholds.[3,5,7,8] It is now being understood that the degree of hypergylcemia and the duration of uncontrolled hyperglycemia is more important than the duration of the disease itself.

Screening of all patients with diabetes for hearing loss in a longitudinal study may provide a clearer understanding of the relationship between diabetes and hearing loss. It is the intention of the author to continue with such a study and evaluate the function of outer hair cells in a well selected diabetic population in India.

## Conclusions

In this study, the auditory acuity of hyperglycemic and normoglycemic subjects were studied. The variables influencing the auditory acuity were statistically analyzed which revealed the following:

1. Diabetes mellitus type 2 raises auditory threshold in all frequencies between 250 Hz and 8000 Hz in all age groups in this study.
2. Patients with poor control [HbA1c greater than 8%] of their glycemic status have raised auditory thresholds.
3. The duration of diabetes does not affect auditory thresholds significantly in this study.
4. There was no association between the diabetic age-wise subgroups and their corresponding auditory thresholds. But in the age-wise subgroups in controls, there was an association between the subgroups and auditory thresholds. But the auditory thresholds were still within the normal limits.

These results which show the effect of hyperglycemia on auditory acuity may be explained by diabetic microangiopathy of the inner ear.

## Summary

The relationship between diabetes and hearing loss is controversial. The present study was undertaken to better understand the disease and its influence on hearing acuity. The study involved determination of the auditory thresholds in two groups of subjects namely, hyperglycemic and normoglycemic subjects. The hearing thresholds were evaluated in both the groups using a pure tone audiometer (ARPHI 500 MK 1). The glycemic status (FBS, PPBS) and the degree of the glycemic control (HbA1C) of the hyperglycemic subjects were also assessed. The results obtained were then subjected to statistical treatment.

The results showed that the hyperglycemic subjects were significantly hearing impaired than the normoglycemic group. This hearing impairment was noted in all the frequencies tested. The glycemic status and the degree of glycemic control played a significant role in reducing the auditory acuity of the hyperglycemic subjects, while the duration of hyperglycemia and the age of the subjects did not influence the hearing acuity.
